# Supplementary material for: Immunogenicity and In Vivo Protective Effects of Recombinant Nucleocapsid-Based SARS-CoV-2 Vaccine Convacell®
Source: Vaccines (Basel). 2023 Apr 20;11(4):874. doi: 10.3390/vaccines11040874 (PMC10141225; doi:10.3390/vaccines11040874)
Supplement: Supplementary file 1 [file vaccines-11-00874-s001.zip › vaccines-2352651-supplementary.pdf]

Supplementary Material

Supplementary Figures

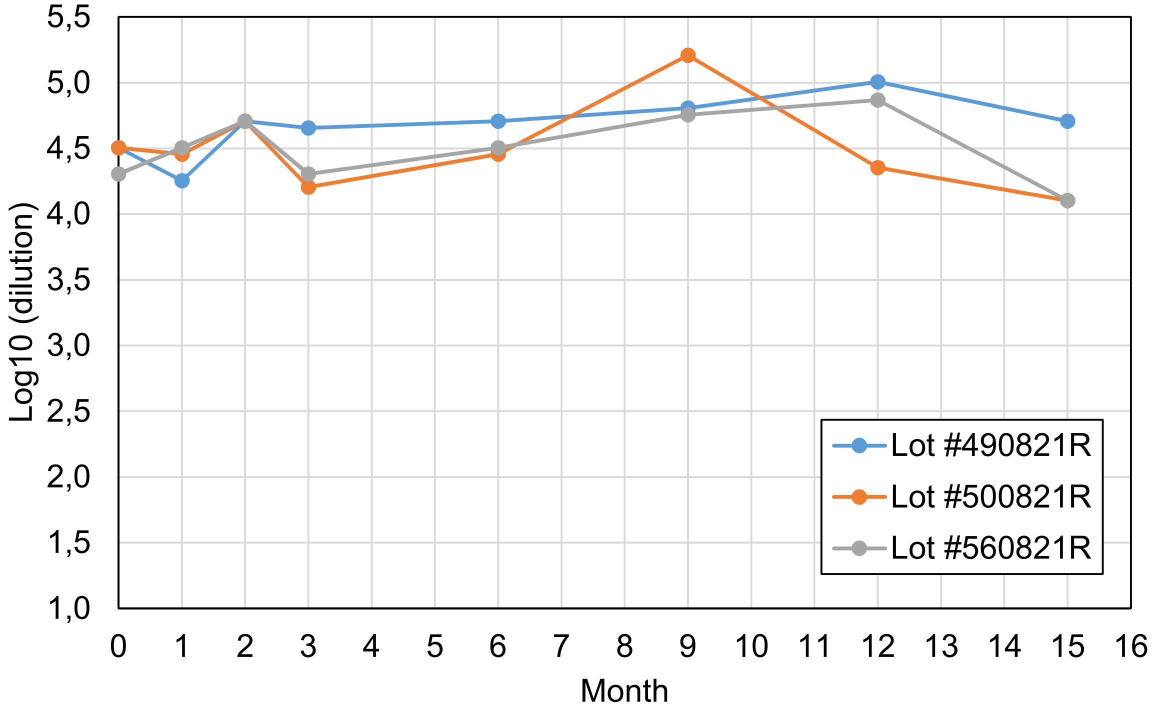

Figure S1. Stability of vaccine Convacell® is confirmed by measurement of N-specific IgG antibodies in serum of mice that were immunized with vaccine samples stored at 2-8°C for 15 months. See Materials and Methods for experiment and ELISA details.

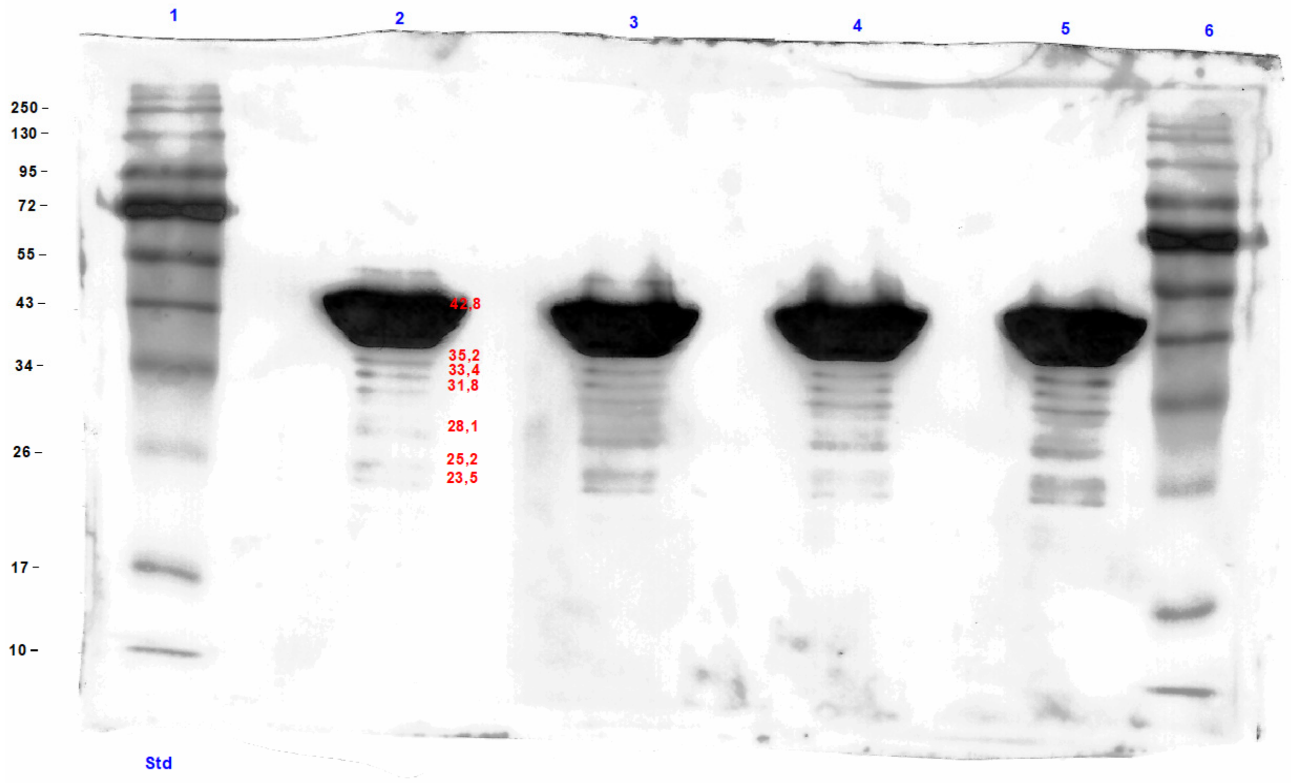

Figure S2. Western blot of recombinant protein N solution, uncropped photo of membrane. Lanes 1 and 6 are molecular weight markers, lines 2-5 are replicate samples of recombinant N protein solution. Molecular weights in kDa of marker bands in lane 1 are indicated in black, molecular weights in kDa of protein bands in lane 2 are indicated in red. Note that the gel is overloaded, and as such, the software analyses the molecular mass of the main protein band – 42.8 kDa as lower than it is. Band 1, with molecular weight of 42.8 kDa has densitometric intensity of 3883984; band 2, with molecular weight of 35.2 kDa, has densitometric intensity of 95424; band 3, with molecular weight of 33.4 kDa, has densitometric intensity of 35784; band 4, with molecular weight of 31.8 kDa, has densitometric intensity of 16756; band 5, with molecular weight of 28.1, has densitomertic intensity of 5112, band 6, with molecular weight of 25.2, has densitometric intensity of 12496; band 6, with molecular weight of 23.5, has densitometric intensity of 4544.

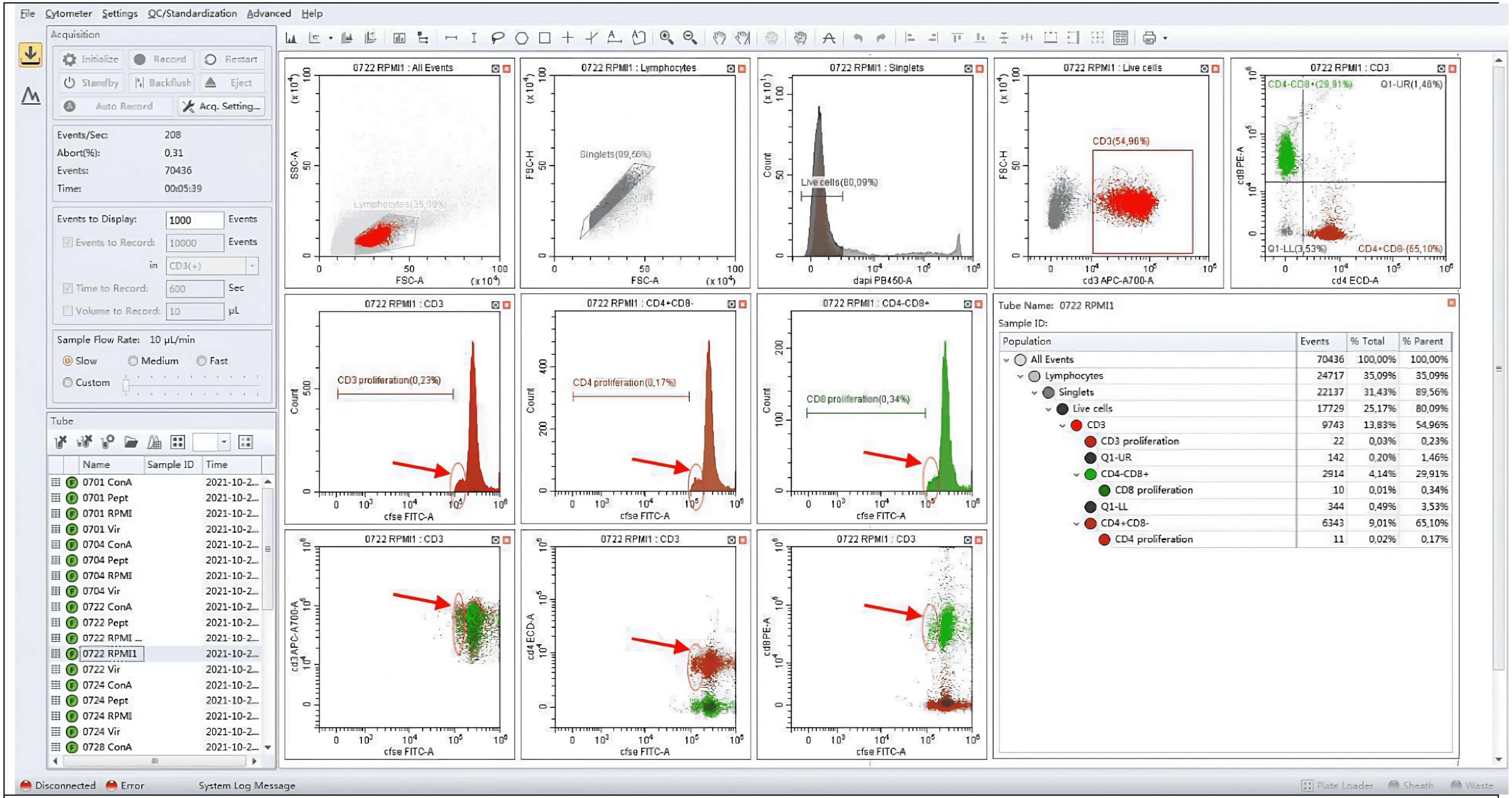

Figure S3. Gating strategy to assess phenotype of proliferating N-specific T-cells in Convacell®-injected marmoset monkeys.

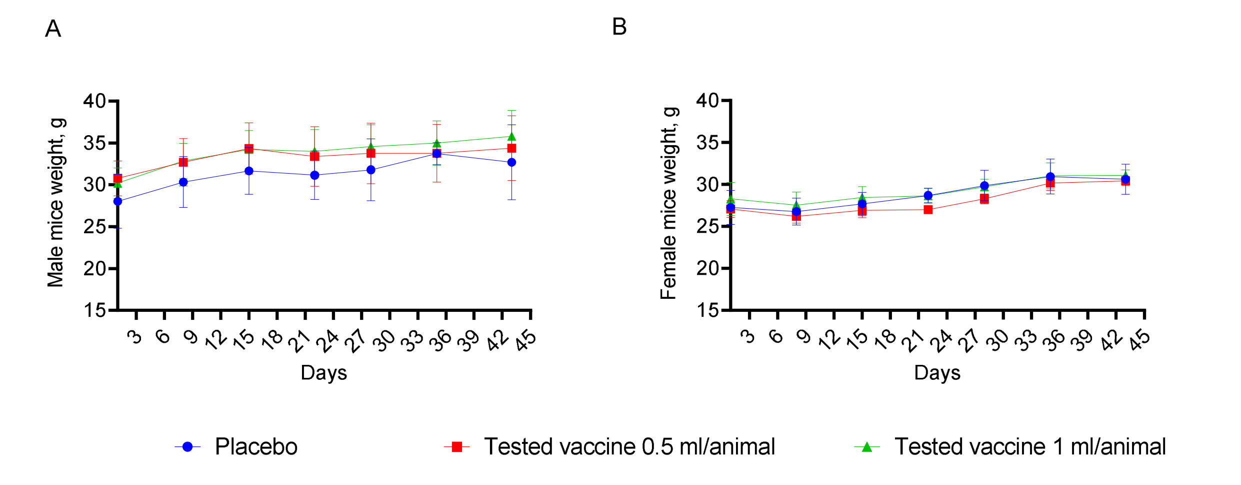

Figure S4. Animal weight of Male (A) and Female (B) Balb/c mice during safety assessment. Results are presented as average mean (M) and standard deviation (SD).

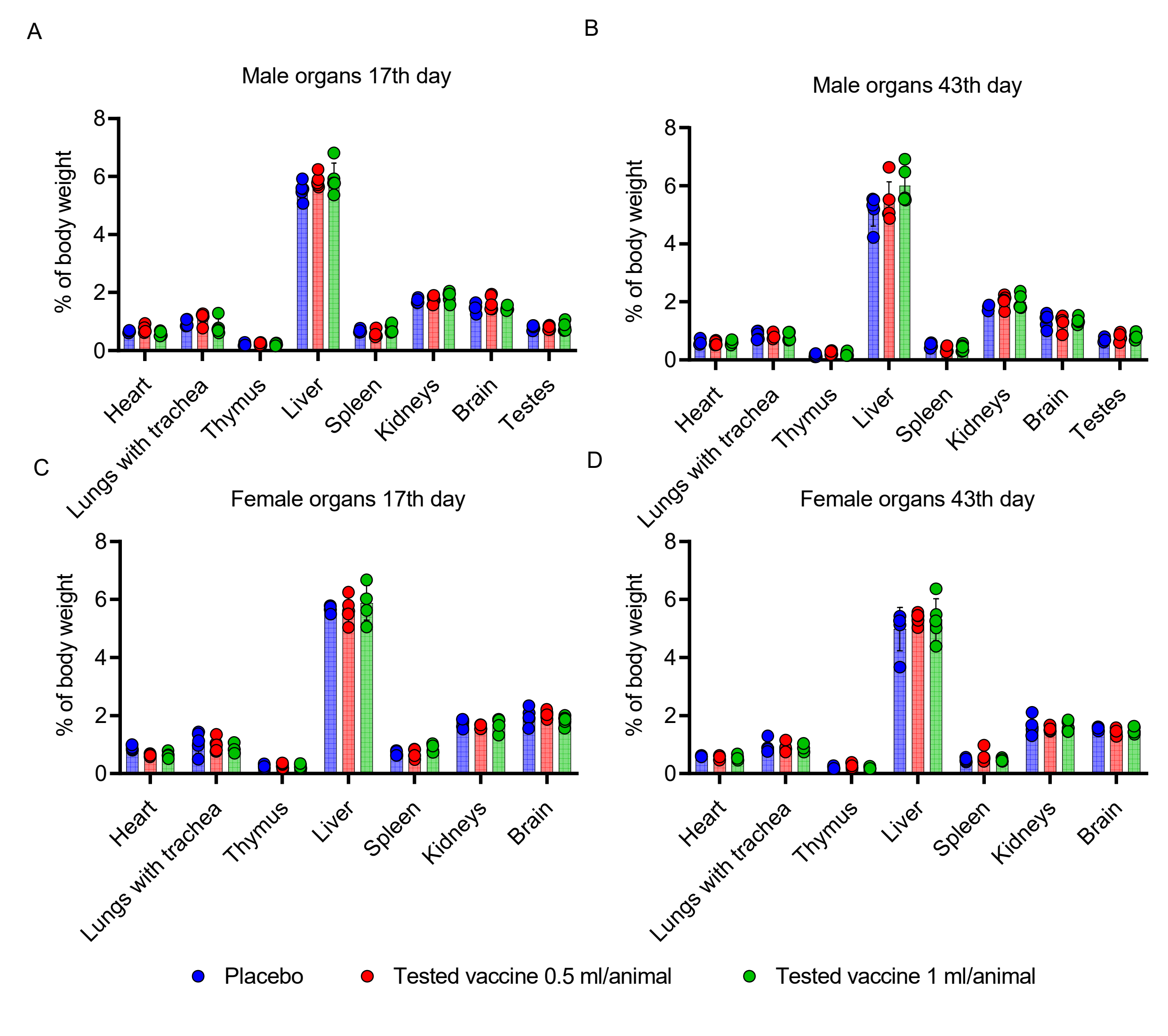

Figure S5. Organ weight as % of body weight of Male (A, B) and Female (B, C) Balb/c mice on safety study days 17 and 43. Results are presented with individual values as average mean (M) and standard deviation (SD).

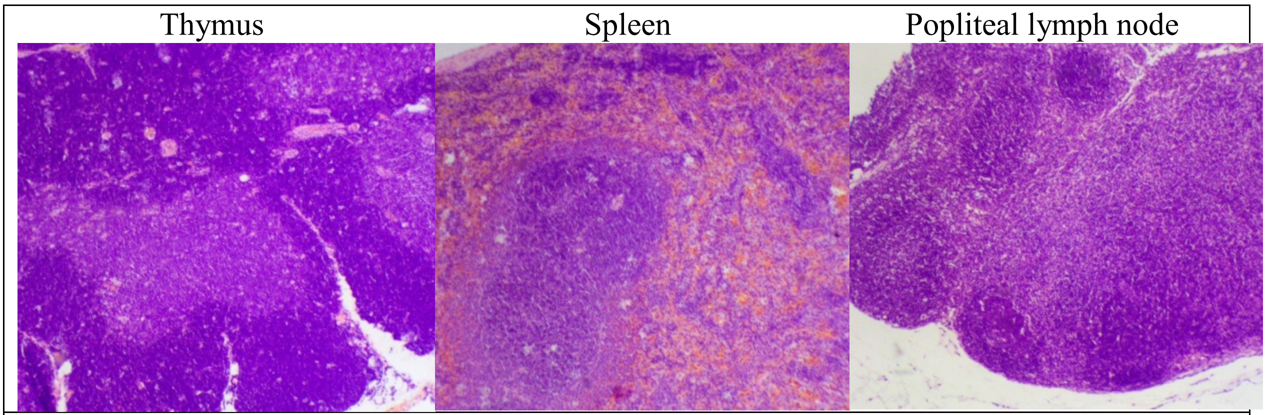

Figure S6. Representative microphotographs of immunocompetent organs of Convacell®-injected male rabbits. Normal microscopic structure. Magnification 40×. Animals were injected thrice on study day 1, 8 and 15.

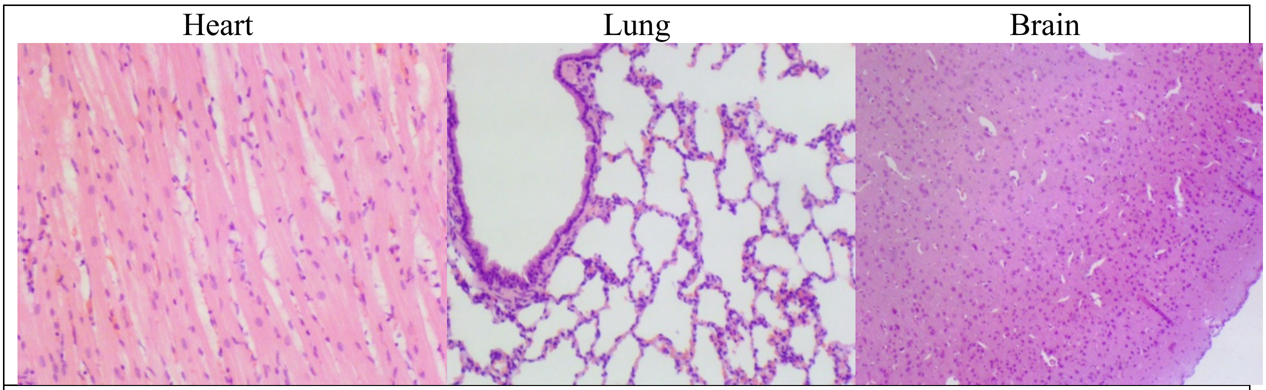

Figure S7. Representative microphotographs of heart, lung and brain of Convacell®-injected male rabbits. Normal microscopic structure. Magnification 40×. Animals were injected thrice on study day 1, 8 and 15.

№	Intact animals	Not vaccinated + infected	Placebo + infected	Vaccinated + infected
1	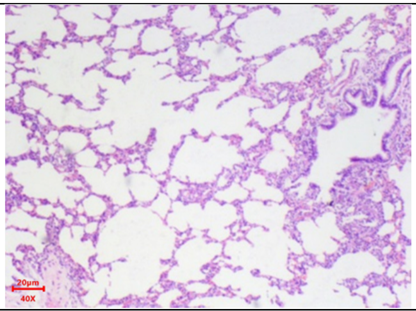	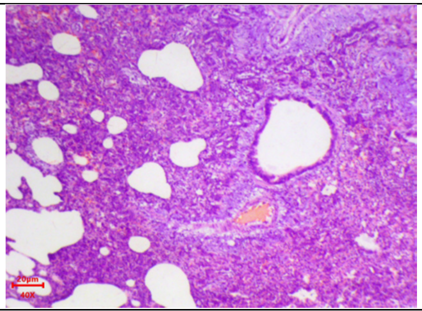	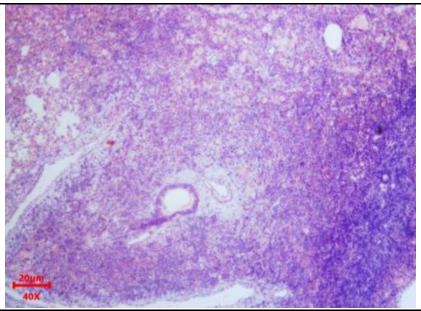	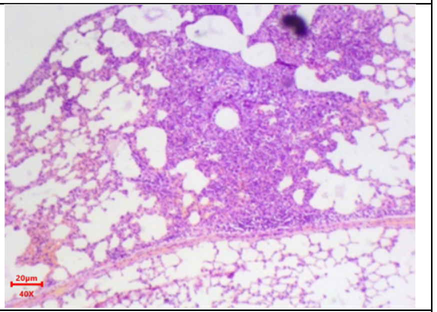
2	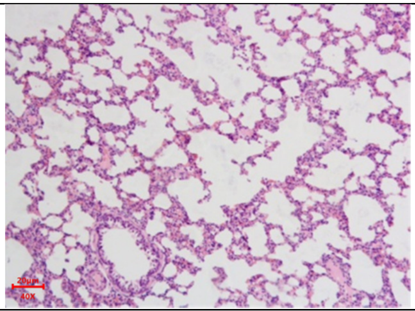	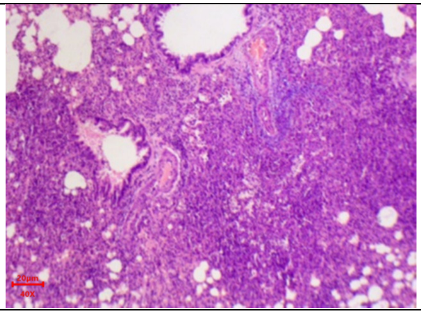	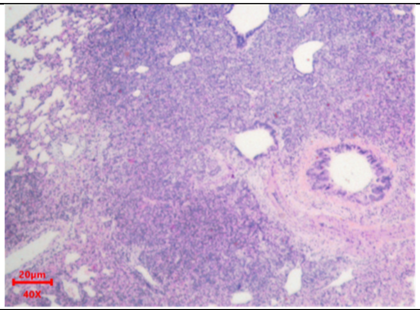	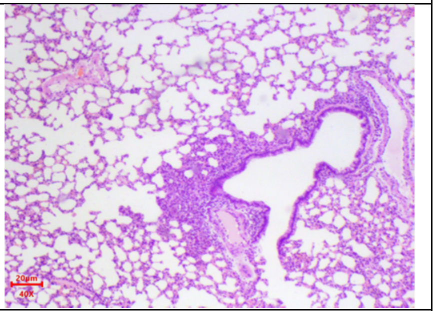
3	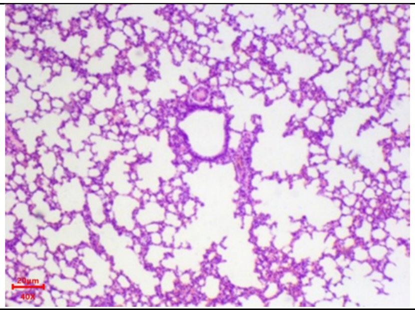	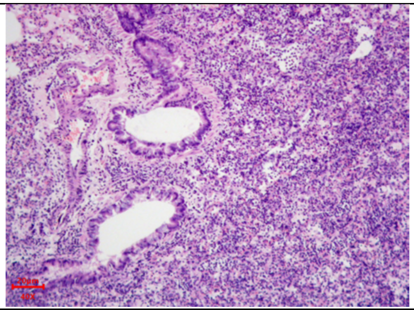	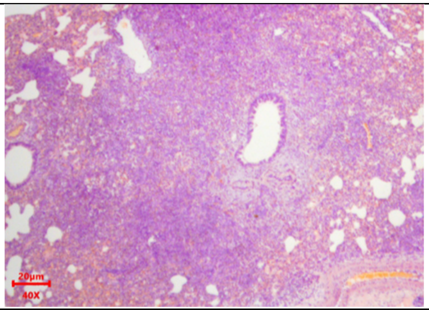	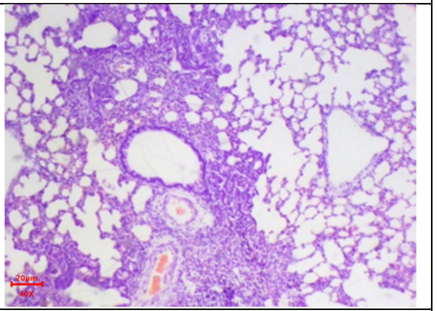

Figure S8. Representative lungs microphotographs of female Syrian hamsters injected with Convacell®. Morphological examination was performed using Axio Scope A1light optical microscope (Carl Zeiss Microscopy GmbH, Germany) at ×40 magnification. Microphotographs were analyzed using AxioCamICc 1 digital camera (Carl Zeiss Microscopy GmbH, Germany) and ZEN 2012 (Germany) software.

№	Intact animals	Not vaccinated + infected	Placebo + infected	Vaccinated + infected
4	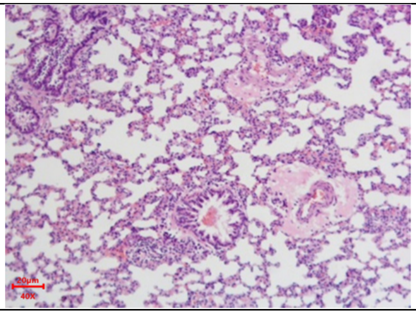	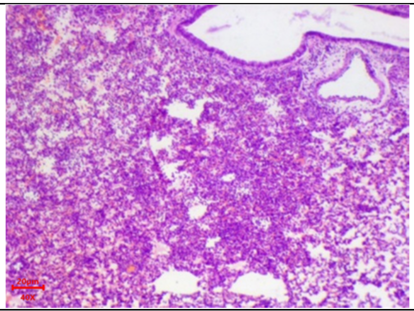	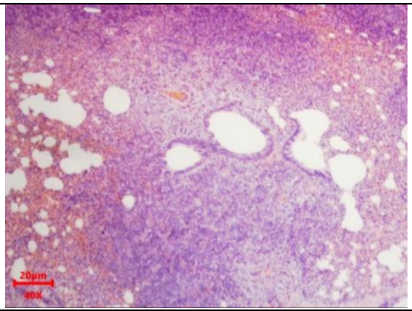	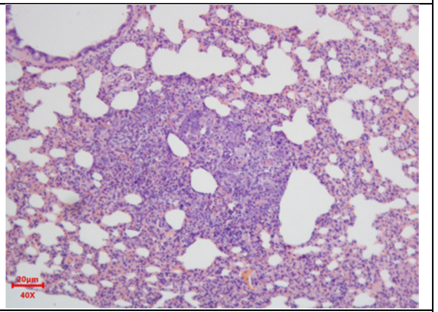
5	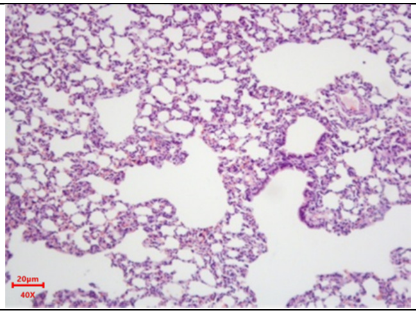	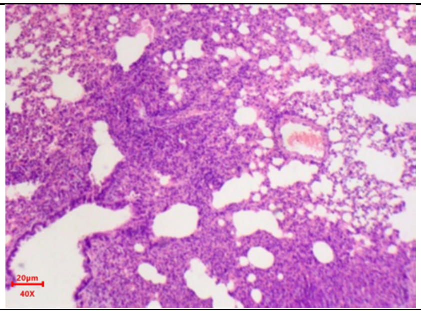	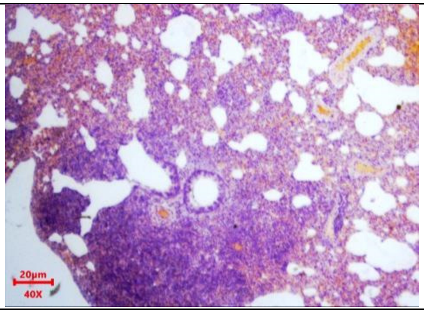	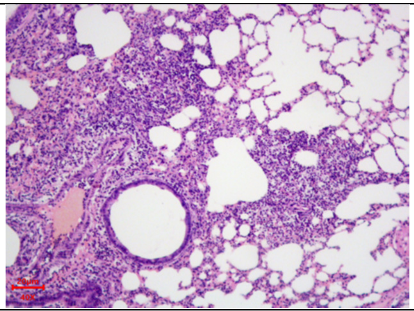

Figure S8. (Continued).

Supplementary Tables

Table S1. Ethics committees and decisions credentials.

Study	Facility	Ethics committee approval
Safety: mice	RMC "Home of pharmacy" JSC, Kuzmolovsky, Leningrad region, Russia, 188663	Decision of the bioethical commission No. 3.24/20 dated May 20, 2020
Safety: rabbits	RMC "Home of pharmacy" JSC, Kuzmolovsky, Leningrad region, Russia, 188663	Decision of the bioethical commission No. 7.24/20 dated May 20, 2020
Immunogenicity: mice	RMC "Home of pharmacy" JSC, Kuzmolovsky, Leningrad region, Russia, 188663	Decision of the bioethical commission No. 2.12/21 dated March 17, 2021
Immunogenicity: hamsters	RMC "Home of pharmacy" JSC, Kuzmolovsky, Leningrad region, Russia, 188663	Decision of the bioethical commission No. 3.12/21 dated March 17, 2021
Immunogenicity: rabbits	RMC "Home of pharmacy" JSC, Kuzmolovsky, Leningrad region, Russia, 188663	Decision of the bioethical commission No. 1.12/21 dated March 17, 2021
Immunogenicity: NSG mice	The Jackson Laboratory – Sacramento facility in vivo services, 1650 Santa Ana Avenue, Sacramento, CA, USA, 95838	Institutional Animal Care and Use Committee approved protocol, August 23, 2021
Immunogenicity: marmoset monkeys	M.P. Chumakov Federal Scientific Center for Research and Development of Immunobiological Drugs, household 8, building 1, Polio Institute settlement, Moscow, Russia, 108819	Ethical Committee of FGANU "FNCIRIP named after M.P. Chumakov RAS" (Institute of Poliomyelitis) (No. 220921-1 dated September 22, 2021).
In vivo protectiveness: hamsters	RMC "Home of pharmacy" JSC, Kuzmolovsky, Leningrad region, Russia, 188663	Decision of the bioethical commission No. 5.12/21 dated March 17, 2021

Table S2. Semi-quantitative lung histological analysis scores used in the Convacell® protectiveness experiment in Syrian hamsters.

<table>
<tr><th>Pathology type</th><th>Marker</th><th>Scoring</th></tr>
<tr><td rowspan="4">Lung inflammation</td><td>Interstitial pneumonia</td><td rowspan="4">0 – none observed
1 – minimal
2 – mild
3 – moderate
4 – severe</td></tr>
<tr><td>Bronchitis</td></tr>
<tr><td>Epithelial necrosis of bronchioles and alveoli</td></tr>
<tr><td>Epithelial alveolar cell hyperplasia type II</td></tr>
<tr><td rowspan="4">Immune cell infiltration</td><td>By neutrophils</td><td rowspan="4">0 – none observed
1 – minimal
2 – mild
3 – moderate
4 – severe</td></tr>
<tr><td>By macrophages</td></tr>
<tr><td>By lymphocytes</td></tr>
<tr><td>Perivascular lymphocytic infiltration</td></tr>
<tr><td rowspan="2">Pulmonary edema</td><td>Alveolar edema</td><td rowspan="2">0 – none observed
1 – minimal
2 – mild
3 – moderate
4 – severe</td></tr>
<tr><td>Perivascular edema</td></tr>
</table>

Table S3. Behavioral and motor activity of male Balb/c mice in open field test on the 16th day of Convacell® safety experiment, Me(Q1;Q3), n=5

№	Group	Dose, ml/animal	Analyzed parameter, counts							
			Visits to the center	Square crossing	Wall rearing	Hole looking	Rearing	Self-grooming	Urinations	Defecations
1	Placebo	1	2.0 (1.0;2.0)	12.0 (8.0;12.0)	0.0 (0.0;1.0)	17.0 (16.0;24.0)	0.0 (0.0;0.0)	1.0 (0.0;1.0)	0.0 (0.0;0.0)	1.0 (0.0;1.0)
2	Convacell®	0.5	2.0 (1.0;2.0)	19.0 (14.0;24.0)	0.0 (0.0;1.0)	22.0 (20.0;26.0)	0.0 (0.0;0.0)	0.0 (0.0;1.0)	0.0 (0.0;0.0)	2.0 (1.0;4.0)
3		1	2.0 (1.0;3.0)	16.0 (14.0;18.0)	0.0 (0.0;2.0)	23.0 (20.0;26.0)	0.0 (0.0;0.0)	1.0 (0.0;1.0)	0.0 (0.0;0.0)	2.0 (1.0;2.0)

Table S4. Behavioral and motor activity of female Balb/c mice in open field test on the 16th day of Convacell® safety experiment, Me(Q1;Q3), n=5

№	Group	Dose, ml/animal	Analyzed parameter, counts							
			Visits to the center	Square crossing	Wall rearing	Hole looking	Rearing	Self-grooming	Urinations	Defecations
1	Placebo	1	1.0 (0.0;2.0)	28.0 (24.0;29.0)	0.0 (0.0;1.0)	34.0 (27.0;36.0)	0.0 (0.0;0.0)	0.0 (0.0;1.0)	0.0 (0.0;0.0)	1.0 (1.0;2.0)
2	Convacell®	0.5	1.0 (0.0;1.0)	29.0 (23.0;32.0)	0.0 (0.0;1.0)	24.0 (24.0;35.0)	0.0 (0.0;0.0)	1.0 (1.0;2.0)	0.0 (0.0;0.0)	2.0 (1.0;2.0)
3		1	1.0 (0.0;1.0)	27.0 (26.0;29.0)	0.0 (0.0;2.0)	34.0 (32.0;36.0)	0.0 (0.0;0.0)	1.0 (0.0;1.0)	0.0 (0.0;0.0)	1.0 (1.0;2.0)

Table S5. Behavioral and Locomotor activity of male Balb/c mice in open field test on the 41st day of Convacell® safety experiment, Me(Q1;Q3), n=5

№	Group	Dose, ml/animal	Analysed parameter, counts							
			Visits to the center	Square crossing	Wall rearing	Hole looking	Rearing	Self-grooming	Urinations	Defecations
1	Placebo	1	1.0 (0.0;1.0)	16.0 (13.0;19.0)	2.0 (1.0;2.0)	26.0 (24.0;27.0)	0.0 (0.0;0.0)	0.0 (0.0;1.0)	0.0 (0.0;1.0)	2.0 (2.0;2.0)
2	Convacell®	0.5	1.0 (0.0;1.0)	14.0 (13.0;20.0)	0.0 (0.0;1.0)	17.0 (16.0;20.0)	0.0 (0.0;0.0)	1.0 (0.0;1.0)	0.0 (0.0;0.0)	1.0 (1.0;2.0)
3		1	1.0 (0.0;1.0)	19.0 (12.0;22.0)	1.0 (0.0;1.0)	25.0 (21.0;27.0)	0.0 (0.0;0.0)	1.0 (0.0;1.0)	0.0 (0.0;0.0)	1.0 (0.0;2.0)

Table S6. Behavioral and Locomotor activity of female Balb/c mice in open field test on the 41st day of Convacell® safety experiment, Me(Q1;Q3), n=5

№	Group	Dose, ml/animal	Analyzed parameter, counts							
			Visits to the center	Square crossing	Wall rearing	Hole looking	Rearing	Self-grooming	Urinations	Defecations
1	Placebo	1	1.0 (1.0;1.0)	38.0 (31.0;38.0)	2.0 (1.0;2.0)	36.0 (36.0;37.0)	0.0 (0.0;0.0)	1.0 (0.0;1.0)	0.0 (0.0;0.0)	1.0 (1.0;1.0)
2	Convacell®	0.5	0.0 (0.0;1.0)	34.0 (32.0;37.0)	0.0 (0.0;1.0)	32.0 (28.0;39.0)	0.0 (0.0;1.0)	0.0 (0.0;0.0)	0.0 (0.0;0.0)	0.0 (0.0;1.0)
3		1	1.0 (0.0;1.0)	34.0 (31.0;41.0)	1.0 (0.0;1.0)	40.0 (34.0;41.0)	0.0 (0.0;1.0)	0.0 (0.0;0.0)	0.0 (0.0;1.0)	0.0 (0.0;1.0)

Table S7. Hematological parameters of male rabbits used in the Convacell® safety experiment (17th day), M±SD, n=4

Analyzed parameters	Group		
	Control group	Vaccinated	
	1.0 ml/animal	0.5 ml/animal	1.0 ml/animal
WBC Leukocytes, 10^9/l	6.5±1.58	6.6±1.64	8.1±1.20
LYM Lymphocytes, %	51.4±7.54	59.9±6.50	61.0±3.33
MON Monocytes, %	4.1±1.04	2.8±0.13	4.0±0.95
GRA Granulocytes, %	44.5±7.81	37.4±6.53	35.0±4.05
LYM Lymphocytes	3.3±0.75	3.9±0.67	5.0±0.90
MON Monocytes	0.3±0.06	0.2±0.05	0.3±0.13
GRA Granulocytes	3.0±1.15	2.5±1.07	2.8±0.38
RBC Erythrocytes, 10^{12}/l	5.6±0.56	5.8±0.11	5.8±0.41
HGB Hemoglobin, g/l	110.3±9.07	118.8±4.92	117.3±7.41
HCT Hematocrit, %	0.4±0.03	0.4±0.01	0.4±0.03
PLT Thrombocytes, 10^9/l	525.3±124.70	493.3±51.25	492.0±49.99

Table S8. Hematological parameters of female rabbits used in the Convacell® safety experiment (17th day), M±SD, n=4

Analyzed parameters	Group		
	Control group	Vaccinated	
	1.0 ml/animal	0.5 ml/animal	1.0 ml/animal
WBC Leukocytes, 10^9/l	6.2±0.75	5.4±1.24	6.2±0.85
LYM Lymphocytes, %	57.7±7.39	58.2±2.35	59.2±5.62
MON Monocytes, %	3.0±0.59	3.5±0.61	3.2±0.63
GRA Granulocytes, %	39.3±7.13	38.3±2.46	37.7±5.46
LYM Lymphocytes	3.5±0.19	3.1±0.67	3.7±0.55
MON Monocytes	0.2±0.05	0.2±0.05	0.2±0.00
GRA Granulocytes	2.5±0.66	2.1±0.54	2.4±0.55
RBC Erythrocytes, 10^{12}/l	5.3±0.28	5.0±0.10	5.3±0.07
HGB Hemoglobin, g/l	105.8±4.50	100.8±3.20	108.0±6.06
HCT Hematocrit, %	0.4±0.02	0.3±0.01	0.4±0.02
PLT Thrombocytes, 10^9/l	527.3±110.86	626.5±169.22	485.8±96.25

Table S9. Hematological parameters of male rabbits used in the Convacell® safety experiment (43th day), M±SD, n=4

Analyzed parameters	Group		
	Control group	Vaccinated	
	1.0 ml/animal	0.5 ml/animal	1.0 ml/animal
WBC Leukocytes, 10^9/l	7.0±1.28	6.5±0.79	5.8±1.23
LYM Lymphocytes, %	64.2±5.20	66.0±4.80	70.5±5.26
MON Monocytes, %	2.7±0.62	2.9±0.66	3.4±1.20
GRA Granulocytes, %	33.1±5.20	31.2±4.46	26.2±5.31
LYM Lymphocytes	4.5±0.53	4.03±0.48	4.1±1.07
MON Monocytes	0.2±0.05	0.2±0.08	0.2±0.08
GRA Granulocytes	2.4±0.79	2.0±0.42	1.5±0.22
RBC Erythrocytes, 10^{12}/l	6.0±0.50	6.3±0.46	5.7±1.55
HGB Hemoglobin, g/l	126.5±4.80	131.5±4.20	124.5±19.74
HCT Hematocrit, %	0.4±0.02	0.4±0.01	0.4±0.06
PLT Thrombocytes, 10^9/l	395.0±90.63	368.5±30.07	394.8±100.47

Table S10. Hematological parameters of male rabbits used in the Convacell® safety experiment (43th day), M±SD, n=4

Analyzed parameters	Group		
	Control group	Vaccinated	
	1.0 ml/animal	0.5 ml/animal	1.0 ml/animal
WBC Leukocytes, 10^9/l	6.8±1.86	5.4±0.74	4.5±0.81
LYM Lymphocytes, %	65.6±2.50	59.7±5.62	61.2±5.90
MON Monocytes, %	2.5±0.57	3.2±1.08	3.7±0.54
GRA Granulocytes, %	31.9±2.25	37.2±5.59	35.1±5.63
LYM Lymphocytes	4.4±1.26	3.3±0.59	2.8±0.48
MON Monocytes	0.2±0.00	0.2±0.06	0.2±0.05
GRA Granulocytes	2.2±0.61	2.0±0.42	1.6±0.41
RBC Erythrocytes, 10^{12}/l	5.9±0.27	5.9±0.39	5.9±0.73
HGB Hemoglobin, g/l	116.3±6.99	117.3±4.65	121.3±13.48
HCT Hematocrit, %	0.4±0.03	0.4±0.01	0.4±0.04
PLT Thrombocytes, 10^9/l	382.8±180.14	429.8±79.10	444.3±103.69

Table S11. Blood biochemical male rabbits used in the Convacell® safety experiment (17th day), M±SD, n=4

Analyzed parameters	Group		
	Control group	Vaccinated	
	1.0 ml/animal	0.5 ml/animal	1.0 ml/animal
Albumin (A), g/l	45.2±1.43	49.8±3.84	49.6±4.49
Alkaline phosphatase, U/l	299.0±45.66	318.3±22.68	265±35.77
ALT, U/l	39.7±16.63	30.0±19.92	25.3±9.25
AST, U/l	35.6±32.92	23.0±12.75	27.2±20.82
Cholesterol, mM	1.8±0.64	1.6±0.29	1.3±0.26
Creatinine, µM	94.3±9.84	109.8±11.27	115±15.28
Glucose, mM	9.3±0.29	10.0±0.10	10.2±0.97
LDH, U/l	584.3±163.55	402.5±101.24	582.3±211.86
Total protein, g/l	63.0±2.15	66.7±3.43	68.2±5.97
Globulins (G), g/l	17.8±2.12	16.9±0.70	18.6±1.50
A/G ratio	2.6±0.36	3.0±0.32	2.7±0.05
Triglycerides, mM	1.5±0.41	1.4±0.08	1.0±0.18
Urea, mM	4.3±1.63	3.8±0.95	6.3±0.92
Total bilirubin, µM	1.3±0.18	1.4±0.29	2.0±0.33

ALT – alanine aminotransferase, AST – aspartate aminotransferase, LDH – lactate dehydrogenase

Table S12. Blood biochemical female rabbits used in the Convacell® safety experiment (17th day), M±SD, n=4

Analyzed parameters	Group		
	Control group	Vaccinated	
	1.0 ml/animal	0.5 ml/animal	1.0 ml/animal
Albumin (A), g/l	46.7±1.44	46.4±3.35	45.0±1.52
Alkaline phosphatase, U/l	315.0±77.61	271.0±38.95	275.3±35.72
ALT, U/l	26.7±6.95	28.0±6.94	22.0±4.51
AST, U/l	23.3±11.93	16.6±6.37	16.8±8.00
Cholesterol, mM	1.6±0.45	1.6±0.16	1.6±0.31
Creatinine, μM	97.9±5.04	100.2±11.03	97.0±12.33
Glucose, mM	9.3±0.75	9.2±0.36	9.2±0.31
LDH, U/l	629.8±125.24	358.3±66.04	435.8±63.74
Total protein, g/l	65.3±1.78	63.9±1.58	60.8±2.41
Globulins (G), g/l	18.6±2.19	17.4±2.12	15.8±2.23
A/G ratio	2.5±0.35	2.7±0.58	2.9±0.45
Triglycerides, mM	1.3±0.25	1.4±0.25	0.7±0.14
Urea, mM	4.8±1.79	5.3±0.28	3.9±0.69
Total bilirubin, μM	2.1±0.41	1.9±1.08	1.2±0.52

ALT – alanine aminotransferase, AST – aspartate aminotransferase, LDH – lactate dehydrogenase

Table S13. Blood biochemical parameters of male rabbits used in the Convacell® safety experiment (43th day), M±SD, n=4

Analyzed parameters	Group		
	Control group	Vaccinated	
	1.0 ml/animal	0.5 ml/animal	1.0 ml/animal
Albumin (A), g/l	43.3±1.22	45.5±2.78	44.8±0.66
Alkaline phosphatase, U/l	186.0±49.22	363.5±134.18	246.3±35.98
ALT, U/l	50.2±20.23	47.2±13.28	39.7±22.61
AST, U/l	39.5±23.55	37.2±20.14	24.0±3.51
Cholesterol, mM	1.3±0.47	1.6±0.45	1.4±0.36
Creatinine, µM	121.0±19.59	126.2±33.31	124.0±21.73
Glucose, mM	7.3±0.27	7.7±0.42	7.1±0.56
LDH, U/l	399.3±220.12	392.3±133.42	279.8±155.42
Total protein, g/l	61.5±6.85	61.2±1.49	59.2±1.50
Globulins (G), g/l	18.2±7.46	15.7±2.84	14.4±1.18
A/G ratio	2.7±1.03	3.0±0.66	3.1±0.27
Triglycerides, mM	0.8±0.18	0.7±0.30	0.5±0.18
Urea, mM	6.0±1.07	7.5±0.65	6.4±0.57
Total bilirubin, µM	1.5±0.28	1.0±0.15	1.4±0.33

ALT – alanine aminotransferase, AST – aspartate aminotransferase, LDH – lactate dehydrogenase

Table S14. Blood biochemical parameters of female rabbits used in the Convacell® safety experiment (43th day), M±SD, n=4

Analyzed parameters	Group		
	Control group	Vaccinated	
	1.0 ml/animal	0.5 ml/animal	1.0 ml/animal
Albumin (A), g/l	42.6±0.90	42.4±3.18	46.9±2.21
Alkaline phosphatase, U/l	266.5±98.59	262.3±38.25	227.5±52.22
ALT, U/l	43.5±14.03	38.0±3.12	38.3±5.49
AST, U/l	28.1±5.02	24.6±7.02	19.6±2.92
Cholesterol, mM	1.7±0.31	1.9±0.28	2.9±10.00
Creatinine, µM	119.0±15.55	122.4±17.37	135.6±19.44
Glucose, mM	8.0±0.96	7.3±0.73	8.4±0.63
LDH, U/l	385.5±93.44	450.8±135.88	426.8±73.42
Total protein, g/l	57.4±2.31	56.7±70.00	59.7±4.43
Globulins (G), g/l	14.8±1.52	14.3±4.17	12.8±2.52
A/G ratio	2.9±0.25	3.1±0.69	3.7±0.60
Triglycerides, mM	0.7±0.11	0.8±0.28	0.7±0.16
Urea, mM	6.2±0.73	6.3±1.51	6.2±0.94
Total bilirubin, µM	1.4±0.26	1.3±0.43	1.2±0.34

ALT – alanine aminotransferase, AST – aspartate aminotransferase, LDH – lactate dehydrogenase

Table S15. Electrocardiogram parameters of male rabbits used in the Convacell® safety experiment (15th day) , M±SD, n=4

Group	Dose	Electrocardiogram parameters					
		Heart rate beats/min	RR, msec	P, msec	PQ, msec	QRS, msec	QT, msec
Placebo	1.0 ml/animal	239± 119.4	266± 132.8	45± 22.3	77± 38.6	27± 13.6	152± 76.0
Convacell®	0.5 ml/animal	230± 115.1	269± 134.4	37± 18.6	74± 36.8	29± 14.6	152± 75.8
	1.0 ml/animal	216± 107.9	298± 148.9	39± 19.3	73± 36.5	30± 14.8	155± 77.4

Table S16. Electrocardiogram parameters of female rabbits used in the Convacell® safety experiment (16th day) , M±SD, n=4

Group	Dose	Electrocardiogram parameters					
		Heart rate beats/min	RR, msec	P, msec	PQ, msec	QRS, msec	QT, msec
Placebo	1.0 ml/animal	238± 118.8	258± 128.8	49± 24.5	74± 37	32± 15.9	123± 61.6
Convacell®	0.5 ml/animal	223± 111.6	271± 135.6	44± 22.1	82± 41	30± 14.9	146± 72.8
	1.0 ml/animal	233± 116.5	259± 129.4	47± 23.5	81± 40.5	30± 15.1	141± 70.6

Table S17. Arterial blood pressure of male and female rabbits used in the Convacell® safety experiment (15th and 16th days), mmHg M±SD, n=4

Group	Dose	Male		Female	
		SAP	DAP	SAP	DAP
Control group (Placebo)	1.0 ml/animal	124±62.0	70±35.1	106±53.1	51±25.6
Vaccinated	0.5 ml/animal	111±55.5	66±33.1	105±52.5	57±28.3
	1.0 ml/animal	106±53.1	58±28.9	116±58.1	55±27.3

SAP – systolic arterial pressure, DAP – diastolic arterial pressure

Table S18. Respiratory rate of male and female rabbits used in the Convacell® safety experiment (15th and 16th days), n=4

Group	Dose	Respiratory rate, breaths per minute	
		Male	Female
Placebo	1.0 ml/animal	70±35.1	69±34.3
Vaccinated	0.5 ml/animal	63±31.3	76±38.0
	1.0 ml/animal	57±28.5	66±33.0

Table S19. Results of Convacell® pyrogenicity testing in rabbits

№ of temperature measurement	Temperature*, ºC		
	Animal 1	Animal 2	Animal 3
Before vaccine intravenous injection			
1	38.9	38.9	38.9
2	38.8	38.8	38.8
After vaccine injection with 30 minutes interval			
1	38.7	38.8	38.8
2	38.7	38.8	38.8
3	38.7	38.8	38.8
4	38.7	38.8	38.8
5	38.8	38.8	38.8
6	38.7	38.8	38.8
Δt	0	0	0
∑ Δt	0		

* – the average temperature values obtained from three measurements are presented.

Table S20. Mann-Whitney test with two-stage step-up results for RT-PCR in study of Convacell® protective effect in Syrian hamsters model.

**Group 1**	**Group 2**	**p**	**U**
Placebo	Infected	0.0470	5.0
Vaccine	Infected	0.0079	0.0
Vaccine	Placebo	0.0230	1.0

Table S21. Mann-Whitney test with two-stage step-up results for histology in study of Convacell® protective effect in Syrian hamsters model.

**Group 1**	**Group 2**	**p**	**U**
Infected	Intact	0.0079	0.0
Placebo	Intact	0.0079	0.0
Vaccine	Intact	0.0079	0.0
Placebo	Infected	0.6825	10.0
Vaccine	Infected	0.0238	1.5
Vaccine	Placebo	0.1111	4.5
